# Knockdown of *Tet2* Inhibits the Myogenic Differentiation of Chicken Myoblasts Induced by Ascorbic Acid

**DOI:** 10.3390/ijms232213758

**Published:** 2022-11-09

**Authors:** Yinglin Lu, Kai Shi, Haobin Wang, Heng Cao, Fan Li, Jing Zhou, Minli Yu, Debing Yu

**Affiliations:** Department of Animal Genetics, Breeding and Reproduction, College of Animal Science and Technology, Nanjing Agricultural University, No. 1 Weigang, Nanjing 210095, China

**Keywords:** 5-azacytidine, DNA demethylation, myogenic differentiation, *Tet2*, vitamin C

## Abstract

Ascorbic acid (also called Vitamin C, VC) strengthens the function of Tets families and directly increases DNA demethylation level to affect myogenic differentiation. However, the precise regulatory mechanism of DNA methylation in chicken myogenesis remains unclear. Results of present study showed that the mRNA expression of *MyoD* significantly decreased and *MyoG* and *MyHC* increased in myoblasts treated with 5 μM 5-azacytidine (5-AZA) and 5 μM VC (*p* < 0.05). Results also indicated the formation of myotubes was induced by 5-AZA or VC, but this effect was attenuated after knockdown of *Tet2*. In addition, the protein expression of TET2, DESMIN and MyHC was remarkable increased by the addition of 5-AZA or VC, and the upregulation was inhibited after knockdown of *Tet2* (*p* < 0.05). DNA dot blot and immunofluorescence staining results suggested that the level of 5hmC was significantly increased when treated with 5-AZA or VC, even by *Tet2* knockdown (*p* < 0.05). Moreover, 5-AZA and VC reduced the level of dimethylation of lysine 9 (H3K9me2) and trimethylation of lysine 27 of histone 3 (H3K27me3), and this inhibitory effect was eliminated after *Tet2* knockdown (*p* < 0.05). These data indicated that *Tet2* knockdown antagonized the increased levels of 5hmC and H3K27me3 induced by 5-AZA and VC, and eventually reduced myotube formation by modulating the expression of genes involved in myogenic differentiation. This study provides insights that epigenetic regulators play essential roles in mediating the myogenic program of chicken myoblasts.

## 1. Introduction

Recently, many studies revealed that DNA methylation and histone modification exert essential roles in regulating myogenic differentiation [1,2]. It was reported that the whole genomic DNA methylation decreased during myoblast differentiation [3]. Epigenetic studies increase the knowledge of the molecular mechanism regulating myogenic related-gene expression and myogenesis program [4,5]. Fundamental studies are still required to understand the details of epigenetic regulation in chicken myogenesis.

Myoblasts are myogenic precursor cells that play an important role during skeletal muscle development. The fate of myoblasts is determined by transcription factors including *Pax3*/*Pax7* and the myogenic regulatory factors (MRFs), which work together to establish the epigenetic state in the processes of myofiber formation [6,7]. A series of studies has shown that DNA demethylation exerted a critical role in myogenesis [2,8,9]. The expression of TET1 and TET2 are significantly increased and the DNA methylation level decreases after induction of myoblast differentiation [10]. The *Tet1*/*Tet2* knockout myoblasts showed muscle atrophy and attenuated *MyoD* expression and myotube formation in mice. Interestingly, knockdown of *Tet2* in C2C12 led to increased DNA methylation of gene promoters of MRFs, which correlated with decreased expression of these genes and abrogated myogenic differentiation [10]. Our previous study also indicated that *Tet2* affected chicken myoblast differentiation by regulating DNA methylation and histone methylations [8].

Previous study showed that the demethylation of *MyoD* and *MyoG* promoter was required for myoblast differentiation [11,12]. *MyoG* push the differentiation program forward, accompany with epigenetic regulation leading to MRFs expression and myotube fusion. It was reported that the differentiation capacity of C2C12 was reduced by treatment of sodium arsenic, as a result of the increased DNA methylation and decreased expression of *MyoG* [13]. However, hypermethylation of *MyoG* decreased sharply after the induction of differentiation in C2C12 [14]. In addition, a decrease of DNA methylation of *MyoD* promoter occurred during myoblast differentiation [7,15]. Taken together, these findings showed that transcription factors act though epigenetic regulation to genes are required to ensure myogenetic differentiation.

Several molecules have been identified to facilitate myoblast differentiation during the past studies [16,17,18]. Although its mechanism has not been completely revealed, VC was considered to promote myoblast differentiation probably due to mediate DNA demethylation [16]. Studies have also shown that 5-AZA promoted myogenic differentiation by inhibiting DNA methylation and increasing DNA demethylation [18]. Treatment of mouse myoblasts with 5-AZA affected the expression of cell cycle--related genes and myogenic genes to regulate early differentiation [18]. A previous study showed that VC enhanced the activity of Tets enzyme and rapidly increased the level of 5hmC. This effect was inhibited in *Tet1* and *Tet2* knockout mice, indicating that VC exerted its function by mediating the Tets family [19]. Recent studies found that VC strengthens the function of Tets family and directly increased the level of DNA demethylation to promote myogenic differentiation in mice [10]. A recent study revealed that trimethylation of lysine 9 (H3K9me3) and H3K27me3 are accompanied by chromatin compaction, which is required for myoblast differentiation [8,20]. The demethylation of H3K27me3 was mediated by demethylase KDM6a that opened the chromatin to permit *MyoG* expression and start to differentiation [21,22]. VC deficiency has been reported to decrease the expression of histone demethylases KDM6b, leading to rapid accumulation of H3K27me3 in cells [9]. Therefore, 5-AZA and VC affected the demethylation of DNA and histone to promote myoblast differentiation. This accumulating evidence corroborated the findings that VC and 5-AZA regulated DNA methylation level and induced myoblast differentiation.

Currently, few data are available about DNA methylation status of epigenetic regulation, so the precise epigenetic mechanisms regulating myogenesis in chickens still needs to be further studied. To address this issue, the present study aimed to explore the relationship with VC and *Tet2* in regulation of myogenic differentiation by using a chicken myoblast cell model. *Tet2* knockdown myoblasts were treated with 5-AZA and VC to investigate their inductive effects on myoblast differentiation. Thus, this study will provide valuable information on exploration of the epigenetic mechanism of myogenesis.

## 2. Results

### 2.1. Effect of 5-AZA and VC on Myogenic Related Gene Expression

**Figure 1:**
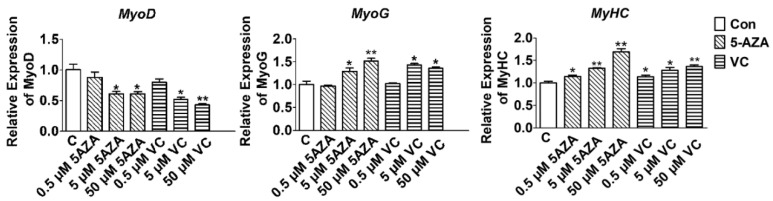
The mRNA expression of myoblast differentiation related genes was analyzed by qRT-PCR. Myoblasts were treated with 5-AZA (0, 0.5, 5 and 50 μM) and VC (0, 0.5, 5, and 50 μM), then the expression of myogenic genes was measured after 3 days. Data are presented as the means ± SEM. *p* < 0.05 is shown as *; *p* < 0.01 is shown as **.

In order to determine the effect of decreased *Tet2* expression on myoblast differentiation induced by 5-AZA and VC, the optimal concentrations in the regulation of myoblast differentiation were firstly examined. Myoblast differentiation was induced when the density of myoblasts reached 90%. Different concentrations of 5-AZA and VC were added to the differentiation medium, and the change of myogenic factors level was detected after 3 days. Results suggested that 5 and 50 μM 5-AZA remarkably reduced the mRNA expression of *MyoD* and increased the expression of *MyoG* and *MyHC* (*p* < 0.05) (Figure 1). The expression of *MyoD* was significantly decreased at 5 μM VC (*p* < 0.05) and extremely significantly decreased at 50 μM VC (*p* < 0.01) (Figure 1). However, the expression of *MyoG* and *MyHC* was significantly increased after the treatment with 5 μM and 50 μM VC (*p* < 0.05) (Figure 1). There was no difference in the expression of *MyoD* and *MyoG* when the supplemental concentration of was 0.5 μM 5-AZA and 0.5 μM VC (*p* > 0.05). Therefore, 5 μM 5-AZA and 5 μM VC were selected as the optimal concentrations in the following experiments.

### 2.2. Effects of 5-AZA and VC on Myoblast Differentiation after Tet2 Knockdown

**Figure 2:**
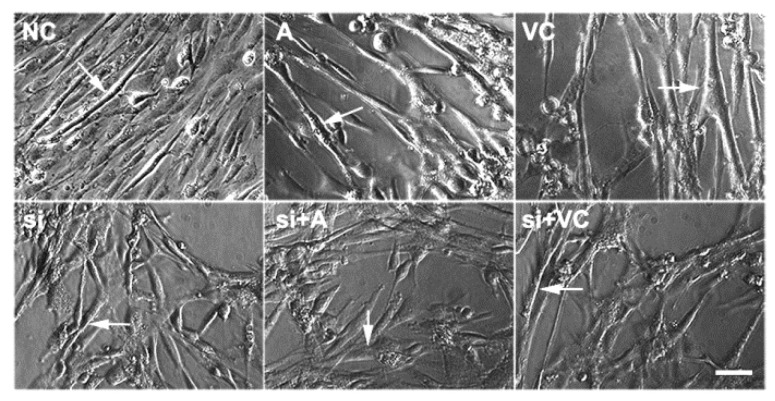
5-AZA and VC facilitated myoblast differentiation. Myoblasts treated with 5-AZA and VC were induced differentiation, and the morphology of myotubes was detected. Arrows indicate differentiated myotubes; Scale bar: 50 μm.

**Figure 3:**
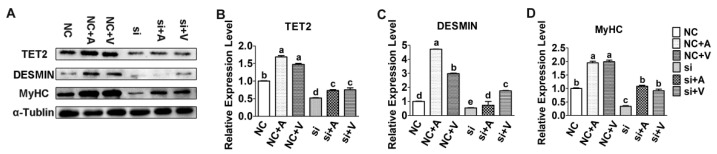
*Tet2* knockdown inhibited myoblast differentiation induced by 5-AZA and VC. (**A**): Protein level of TET2, DESMIN and MyHC was analyzed after *Tet2* knockdown. (**B**–**D**): The gray analysis of relative protein expression. Data are presented as the means ± SEM. Different letters indicate significant differences (*p* < 0.05).

5-AZA and VC induced myoblast differentiation after *Tet2* knockdown was detected. Results showed that the fusion ability of myotubes was enhanced significantly in myoblasts treated with 5-AZA- and VC compared with control, but the formation of myotubes was diminished after *Tet2* knockdown (Figure 2). The protein level of TET2 in myoblasts rose rapidly after treatment with 5-AZA and VC (*p* < 0.01) (Figure 3). It was obviously reduced in the *Tet2* knockdown group, but up-regulated in addition of 5-AZA and VC (*p* < 0.05) (Figure 3). Addition of 5-AZA and VC also significantly increased the protein level of DESMIN and MyHC when compared with the control group (*p* < 0.01) (Figure 3). Moreover, the up-regulation level of DESMIN by 5-AZA was stronger than that of VC. The level of DESMIN was remarkably decreased after *Tet2* knockdown, and this decrease was recovered in addition of 5-AZA and VC (*p* < 0.05) (Figure 3). The level of MyHC was significantly decreased after *Tet2* knockdown, but it quickly returned to the similar lever of control by addition of 5-AZA or VC (*p* < 0.05) (Figure 3). 

### 2.3. Effects of 5-AZA and VC on 5hmC Level after Tet2 Knockdown 

**Figure 4:**
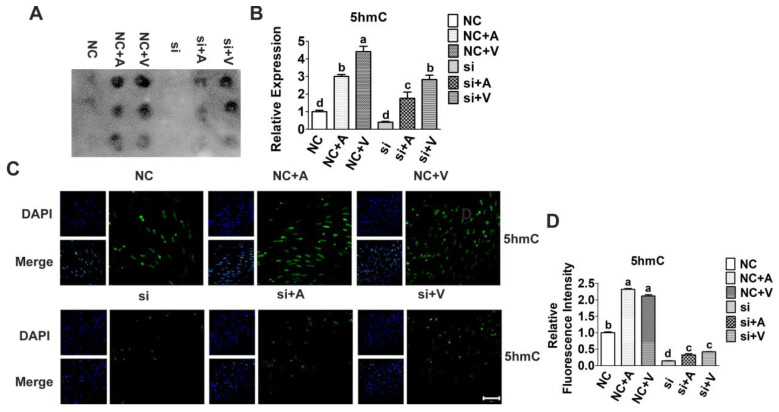
Level of 5hmC in myoblasts treated by 5-AZA and VC after *Tet2* knockdown. The effect of 5-AZA and VC on promoting 5hmC levels after knockdown of *Tet2* was measured by DNA dot blot analysis (**A**,**B**). (**C**,**D**): Immunofluorescence staining for 5hmC (green) on DNA demethylation in myoblasts treated with 5-AZA and VC. The nuclei were counterstained with DAPI (blue). Scale bar = 50 μm. Error bars show mean ± SEM of biological replicates (*n* = 3). Different letters indicate significant difference (*p* < 0.05).

Our previous study demonstrated that *Tet2* knockdown led to a general decrease in 5hmC level and promoted the differentiation of myoblasts. In order to determine whether *Tet2* is required for 5-AZA and VC induced myoblast differentiation, the profile of DNA methylation in *Tet2* knockdown myoblasts treated with/without of 5-AZA and VC were analyzed. Interestingly, result of a DNA dot blot showed that both 5-AZA and VC significantly increased the level of 5hmC (*p* < 0.01) (Figure 4A,B), which suggested that they have similar effects on DNA demethylation. Moreover, it was confirmed by the result of immunofluorescence staining that 5-AZA and VC increased the level of 5hmC compared with the control group (*p* < 0.05) (Figure 4C,D). However, after knockdown of *Tet2*, the level of 5hmC sharply diminished, although addition of 5-AZA and VC restored its level to some extent (*p* < 0.05) (Figure 4). These results indicated that 5-AZA and VC promoted the increase of genomic 5hmC level, but this promotion was inhibited after knockdown of *Tet2*.

### 2.4. Effects of 5-AZA and VC on H3K9me2 and H3K27me3 Level after Tet2 Knockdown 

**Figure 5:**
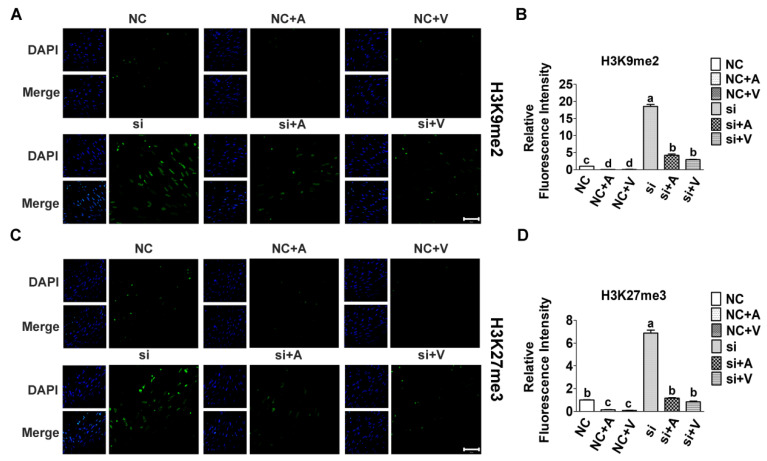
Level of H3K9me2 and H3K27me3 in myoblasts treated by 5-AZA and VC after *Tet2* knockdown. Level of H3K9me2 (**A**,**B**) and H3K27me3 (**C**,**D**) in 5-AZA or VC group was detected by immunofluorescence. (**B**,**D**): Relative fluorescence intensity of H3K9me2 (green) and H3K27me3 (green) was analyzed. The nuclei were counterstained with DAPI (blue). Scale bar: 50 μm. Error bars show mean ± SEM of biological replicates (*n* = 3). Different letters indicate significant difference (*p* < 0.05).

Our previous study showed that the expression of H3K9me2 and H3K27me3 decreased rapidly after myoblast differentiation, and increased significantly after knockdown of *Tet2*. To investigate the effect of 5-AZA and VC on the level of H3K9me2 and H3K27me3, their levels were detected by immunofluorescence staining. Result suggested that the level of H3K9me2 and H3K27me3 were significantly decreased after the addition of 5-AZA and VC compared with control (*p* < 0.05). However, the levels increased rapidly after *Tet2* knockdown, and significantly decreased after 5-AZA and VC treatment (*p* < 0.05) (Figure 5). These results indicated that 5-AZA and VC significantly reduced H3K9me2 and H3K27me3 level, and this inhibition was relieved after *Tet2* knockdown.

### 2.5. Effects of 5-AZA and VC on Histone Methyltransferases Expression after Tet2 Knockdown

**Figure 6:**
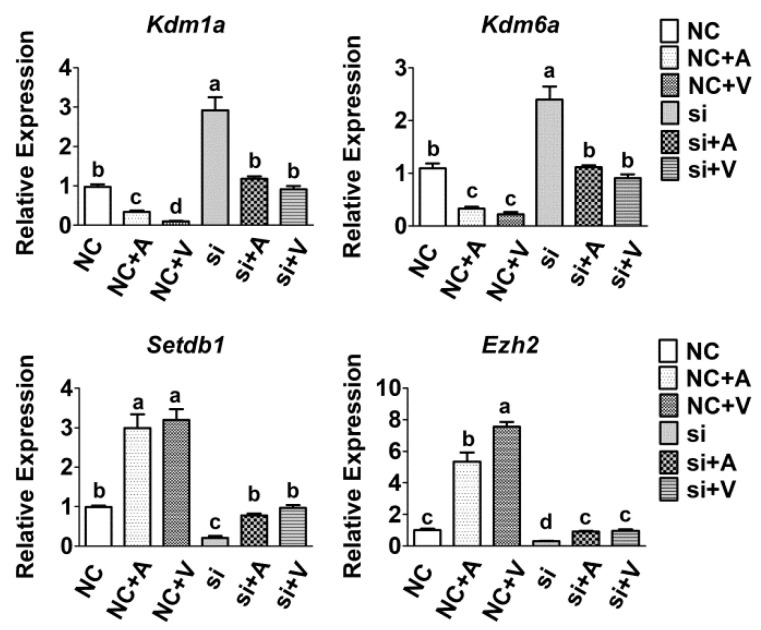
*Tet2* regulated the mRNA expression of histone de/methyltransferases in myoblasts. The expression of histone methyltransferases and demethyltransferases in myoblasts treated by 5-AZA and VC after *Tet2* knockdown was measured by qRT-PCR. Data are presented as the means ± SEM. Different letters indicate significant difference (*p* < 0.05).

To investigate the regulation mechanism of *Tet2* in the level of H3K9me2 and H3K27me3, the mRNA expression of histone methyltransferases and demethyltransferases was measured by qRT-PCR. Result showed that the mRNA expression of histone methyltransferases (*Setdb1* and *Ezh2*) decreased remarkably by treatment of 5-AZA and VC and increased by knockdown of *Tet2* (*p* < 0.05) (Figure 6). However, *Tet2* knockdown induced upregulation was blocked when addition of 5-AZA and VC (*p* < 0.05) (Figure 6). However, the expression level of histone demethyltransferases (*Kdm1a* and *Kdm6a*) was significantly increased when treated with 5-AZA and VC (*p* < 0.05) (Figure 6). By contrast, their expression was obviously decreased after *Tet2* knockdown and recovered by addition of 5-AZA and VC compared to the control group (*p* < 0.05) (Figure 6).

## 3. Discussion

DNA methylation of specific genes is required for myogenic differentiation [21,23]. It was reported that DNA methylation modification was also associated with histone modification during myogenic differentiation. They functioned to allowing the maintenance of myogenic-related gene silencing by DNA methylation [24]. The precise role of epigenetic regulators during myogenic differentiation is still not fully understood. Elucidating the relationship between VC and myoblast differentiation would be important to understand the regulatory mechanism of myogenesis. In the present study, to gain insights into mechanisms by which VC contributes to myoblast differentiation, the genome-wide DNA methylation levels were measured after *Tet2* knockdown by using chicken myoblasts cell model.

Studies showed that the addition of VC into C2C12 up-regulated the expression of Mrf4 and promoted myoblast differentiation and the formation of multinucleated myotubes [25]. This study found that the mRNA levels of *MyoG* and *MyHC* were significantly increased when addition of 5 μM 5-AZA and 5 μM VC. Subsequent results showed that 5-AZA and VC up-regulated the protein level of TET2, MyHC and DESMIN, but the promotion effects were inhibited after *Tet2* knockdown. Combined with the results of previous studies, we speculated that 5-AZA and VC up-regulated the expression of *Tet2* and 5hmC level to promoted myogenic differentiation.

Epigenetic regulation including DNA methylation and histone modification has been widely studied in muscle development. A series of studies emphasized that the epigenetic states are in dynamic change during the process of myogenesis [3,4]. The decline of *Tet2* has been reported to change the status of DNA mythelation and down-regulate myogenic gene expression [8,10]. As demethylation is potently induced in actively differentiating myoblasts, a high level of 5hmC in genomic DNA was observed. Some studies have revealed that VC regulates histone demethylation and modulates related gene expression [26]. This study presented that VC induced genomic DNA demethylation and reduced H3K27me3 level to promote myogenic differentiation by targeting *Tet2*. The decline of DNA methylation and H3K27me3 induced by VC was increased after *Tet2* knockdown, which proved that knockdown *Tet2* antagonizes VC. Furthermore, *Tet2* knockdown-induced upregulation of histone methyltransferases was attenuated by addition of 5-AZA and VC. The expression levels of histone demethyltransferases was obviously decreased after *Tet2* knockdown and recovered by addition of 5-AZA and VC. Therefore, our results using primary-cultured chicken myoblats showed myoblast differentiation was enhanced by addition of VC, while this promotion effect was functionally deteriorated by knockdown of *Tet2*. 

An analogous function of VC with 5-AZA has been observed during myoblast differentiation. As a DNA demethylation reagent, the role of 5-AZA in DNA demethylation has been intensively studied. Previous study found that 5-AZA regulated the cell cycle and MRFs synthesis, and the cell cycle arrest related genes were down-regulated and *MyHC* expression was down-regulated in the 5-AZA group during differentiation [18]. Studies have found that 5-AZA treatment in Hela cells effectively silenced *Dnmt1* transcription and improved the expression of *Tet2* and 5hmC level [27]. The decrease expression of *Tet2* and *Tet3* in hepatocellular carcinoma cells was accompanied by the decrease level of 5hmC, and they were up-regulated in the addition of 5-AZA [28]. These results suggested that 5-AZA played roles in various cell types by promoting the expression of Tets family and up-regulating the level of 5hmC. In the present study, exposure of 5-AZA increased TET2 protein expression, leading to change the genomic methylation state of myoblasts. In addition, VC improved the oxidation of 5mC mediated by Tets family and promoted DNA demethylation in mammals [19]. For example, VC promoted the activity of Tets in ESCs, leading to a rapid increase of 5hmC [19]. Previous studies have found that VC promoted the differentiation of skeletal muscle cells to form myotubes in mice and human, and subsequent studies have found that VC promoted the differentiation of satellite cells [16,25]. To investigate how VC interplays with *Tet2*, the function of VC in *Tet2* knockdown myoblasts was tested in this study. It was found that VC could partially rescue the inhibition of myoblast differentiation in *Tet2* knockdown cells, as a result of increasing myotube formation. Moreover, our result demonstrated that the 5hmC level in *Tet2* knockdown cells was dramatically increased by VC compared to no VC group, even higher than that in NC group. These results suggested that VC relieved the effect of *Tet2* knockdown on reducing the generation of 5hmC, which due to VC enhanced the residual TET2 protein activity caused by incomplete *siTtet2*. 

**Figure 7:**
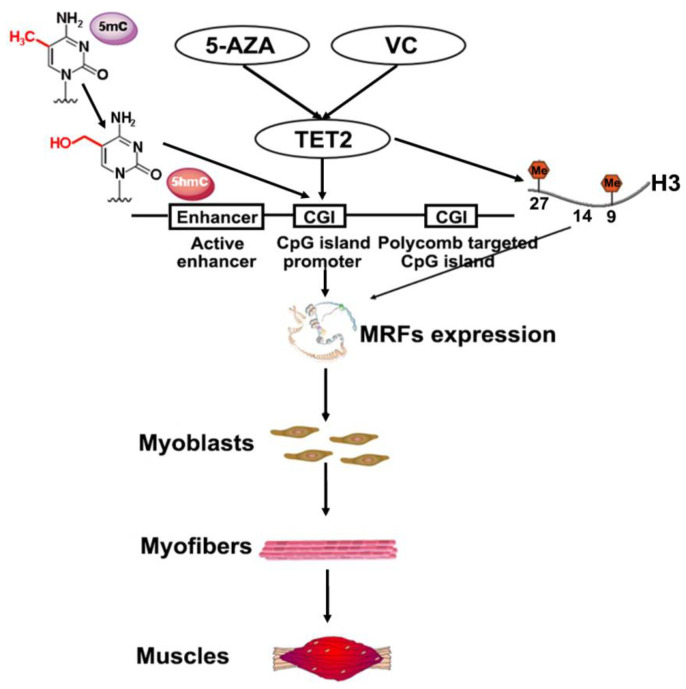
Model of TET2 in regulating myoblast differentiation.

Collectively, the above results indicated that 5-AZA and VC upregulated expression levels of TET2 and MyHC, and knockdown of *Tet2* attenuated the up-regulation of MRFs induced by 5-AZA and VC. 5-AZA and VC exerted remarkable effects on the promotion of DNA demethylation and histone demethylation, but their function was inhibited after *Tet2* knockdown. This evidence suggested that 5-AZA and VC promoted DNA and histone demethylation by regulating *Tet2* expression to achieve myoblast differentiation (Figure 7). Understanding these facts shed insights on the importance of the epigenetic regulation in myogenic program.

## 4. Materials and Methods

### 4.1. Myoblast Cell Culture 

All animal experiments were approved by the Animal Care and Use Committee of Nanjing Agricultural University. Primary myoblast cells were isolated from embryonic 9-day chicken skeletal muscle according to a previous study [17]. Myoblasts were maintained and cultured in growth medium (GM) consisting of DMEM, 10% FBS, and a mixture of 1% penicillin-streptomycin (PS). When the cell confluence reached to 90%, culture medium was replaced with differentiation medium (DM) consisting of DMEM, 2% horse serum (HS, Gibco, Carlsbad, CA, USA) and 1% PS. After induction for 3 days, cells were harvested for further analysis.

### 4.2. The RNA Interference Assays

For RNA interference assays, myoblasts were transfected with *Tet2* siRNA or negative control siRNA using Lipofectamine RNAiMAX reagent (Invitrogen, Carlsbad, CA, USA) according to the manufacturer’s protocal as a previous study [17]. The transfected myoblasts were induced differentiation in addition of 5-AZA and VC.

### 4.3. Quantitative Real-Time PCR (qRT-PCR) Analysis

After culture, total RNA of myoblasts was isolated using Trizol reagent (Invitrogen, Carlsbad, CA, USA) and was reverse transcribed using PrimeScript RT Master Mix (Takara, Dalian, China). Then, qRT-PCR analysis was performed using SYBR Premix ExTaq II (Takara, Dalian, China) with Step-One Real-Time PCR System (Applied Biosystems, Carlsbad, CA, USA). A totol 20 μL reaction mixture contained 2 μL cDNA, 0.5 μL of each primer, 10 μL 2× SYBR Green SuperReal PreMix (ABM Inc., Richmond, BC, Canada), and 7 μL RNase-free H_2_O. PCR amplification was performed as follows: 94 °C for 30 s, followed by 39 cycles at 94 °C for 15 s, 58–62 °C for 30 s. The amount of each transcript was normalized to that of *Gapdh* gene. Results are presented as fold-change using the ^ΔΔ^Ct method. Primer sequences are listed in Table 1.

### 4.4. Western Blotting

Soluble whole-cell lysates of myoblasts were prepared using lysis buffer containing radioimmunoprecipitation assay (RIPA) buffer supplemented with 0.2 nmol/L PMSF (Complete, Roche, Shanghai, China). The lysates were denatured at 95 °C for 5 min and 10 mg of protein samples were subjected to 10% polyacrylamide gel. Blots were transferred to PVDF membranes and incubated in the primary antibodies, including DESMIN (1:50, DSHB, Iowa City, IA, USA), mouse, TET2 (1:400, Abcam, Fremont, CA, USA), MyHC (1:50, DSHB, Iowa City, IA, USA), α-TUBLIN (1:5000, Novus Biologicals Littleton, CO, USA). HRP-conjugated goat anti-rabbit and anti-mouse IgG antibodies (1:5000, Cell Signaling, Beverly, MA, USA) were used as secondary antibodies, respectively. Then, blots were detected using ECL reagents (Thermo Fisher Scientifc, Waltham, MA, USA). The quantity of detected proteins was normalized to that of α-TUBLIN using ImageJ 1.8 software (National Institutes of Health, Bethesda, MD, USA).

### 4.5. Immunocytochemistry

Immunofluorescence staining was performed as previously described [8]. Firstly, the myoblasts were fixed with 4% paraformaldehyde and permeabilized with 0.1% Triton X-100 (Nacalai, Nakagyo-ku, Kyoto, Japan). Then, cells were denatured with 2 M HCl for 30 min followed by neutralised with 100 mM Tris-HCl (PH 8). After blocking in 5% goat serum in TBST for 30 min, cells were immunostained with primary antibodies, including 5hmC (1:500, Active Motif, Carlsbad, CA, USA), H3K9me2 and H9K27me3 (5 μg/mL, Abcam, Fremont, CA, USA). FITC-conjugated secondary antibody (1:1000, KPL Inc., Gaithersburg, MD, USA) were used as secondary antibodies. Cell nuclei were counterstained with 4,6-diaminidino-2-phenylindole (DAPI). Fluorescent images were taken immediately under a confocal laser microscope (Zeiss LSM 700, Carl Zeiss AG, Oberkochen, Germany).

### 4.6. DNA Extraction and Dot Blot Assay

DNA was isolated from cultured myoblasts using a MiniBEST Universal Genomic DNA Extraction Kit (Takara, Dalian, China). Serially diluted 2-fold genomic DNA (300, 150, 75 ng) was spotted onto a nylon membrane (Biosharp, Shanghai, China), following by air-dried and UV-cross linked for 15 min, respectively. After being blocked in 5% skim milk in PBS with 0.1% Tween-20 for 1 h at RT, membranes were incubated with anti-5hmC antibody (1:500; Active Motif, Carlsbad, CA, USA) overnight at 4 ℃. The signal of dots was developed using an ECL reagent (Bio-Rad, Shanghai, China) after incubiation in HRP-conjugated anti-mouse secondary antibody (1:5000; Santa Cruz, Dallas, TX, USA). The signal intensities of dot blot were anylazed by ImageJ 1.8 software.

### 4.7. Statistical Analysis

Each experiment was replicated three times. Data were analyzed by ANOVA and Duncan’s multiple range tests using SPSS 18.0. All data were presented as the means ± SE. Differences at *p* < 0.05 were considered to be statistically significant.

## Figures and Tables

**Table 1:** The primers information.

Gene	Accession No.	Primer Sequence (5′-3′)	Size (bp)
*Gapdh*	NM_204305.1	F: CTGTTGTTGACCTGACCTGC	166
		R:TCAAAGGTGGAGGAAATGGCT	
*MyoD*	NM_204214	F: ATGTCCCATACTGCCTCCAG	235
		R: GTCTTGGAGCTTGGCTGAAC	
*MyoG*	NM_204184	F: GGCTTTGGAGGAGAAGGACT	184
		R: CAGAGTGCTGCGTTTCAGAG	
*MyHC*	NM_001044683	F: GCTTGAACACACTGCAGGAA	236
		R: CTTCAGCCCCTCAGCATAAC	
*Setdb1*	XM_040690848	F: ATCTGAAGGTTGGCATGAGG	170
		R: GGGGTGGTAGTCGTAAGCAA	
*Ezh2*	XM_046912581	F: AGCAAAAAGATCGGGAAGGT	169
		R: GCTCCTGGAAGTTGCTGTTC	
*Kdm1a*	XM_417719	F: GCATTTTGCAAAACCTGGAAT	219
		R: GCAATCACTTCACAGCCTGA	
*Kdm6a*	XM_040662413	F: ATGGAAACGTGCCTTACCTG	151
		R: GGACCTGCCAAATGTGAACT	
